# Locally ablative treatment of breast cancer liver metastases: identification of factors influencing survival (the Mammary Cancer Microtherapy and Interventional Approaches (MAMMA MIA) study)

**DOI:** 10.1186/s12885-015-1499-z

**Published:** 2015-07-14

**Authors:** Max Seidensticker, Benjamin Garlipp, Sophia Scholz, Konrad Mohnike, Felix Popp, Ingo Steffen, Ricarda Seidensticker, Patrick Stübs, Maciej Pech, Maciej PowerskI, Peter Hass, Serban-Dan Costa, Holger Amthauer, Christiane Bruns, Jens Ricke

**Affiliations:** 1 Klinik für Radiologie und Nuklearmedizin, Universitätsklinik Magdeburg, Leipziger Strasse 44, 39120 Magdeburg, Germany; 2 International School of Image-Guided Interventions, Deutsche Akademie für Mikrotherapie, Magdeburg, Germany; 3 Universitätsklinik Magdeburg, Klinik für Allgemein, Viszeral- und Gefäßchirurgie, Magdeburg, Germany; 4 Second Department of Radiology, Medical University of Gdansk, Gdansk, Poland; 5 Institut für Strahlentherapie, Universitätsklinik Magdeburg, Magdeburg, Germany; 6 Universitätsklinik Magdeburg, Universitätsfrauenklinik, Magdeburg, Germany

**Keywords:** Liver metastases, Breast cancer, Oligometastases, Locally ablative therapy, Liver surgery

## Abstract

**Background:**

Liver metastases from breast cancer (LMBC) are typically considered to indicate systemic disease spread and patients are most often offered systemic palliative treatment only. However, retrospective studies suggest that some patients may have improved survival with local treatment of their liver metastases compared to systemic therapy alone. In the absence of randomized trials, it is important to identify patient characteristics indicating that benefit from local treatment can be expected.

**Methods:**

59 patients undergoing radiofrequency ablation (RFA), interstitial brachytherapy (BT), or radioembolization (RE) of LMBC as a salvage treatment were studied. Potential factors influencing survival were analyzed in a multivariate Cox model. For factors identified to have an independent survival impact, Kaplan-Meier analysis and comparison of overall survival (OS) using the log-rank test was performed.

**Results:**

Median OS following local interventional treatment was 21.9 months. Considering only factors evaluable at treatment initiation, maximum diameter of liver metastases (≥3.9 cm; HR: 3.1), liver volume (≥ 1376 mL; HR: 2.3), and history of prior chemotherapy (≥ 3 lines of treatment; HR: 2.5-2.6) showed an independent survival impact. When follow-up data were included in the analysis, significant factors were maximum diameter of liver metastases (≥ 3.9 cm; HR: 3.1), control of LMBC during follow-up (HR: 0.29), and objective response as best overall response (HR: 0.21). Neither the presence of any extrahepatic metastases nor presence of bone metastases only had a significant survival impact. Median OS was 38.7 vs. 16.1 months in patients with metastases < vs. ≥ 3.9 cm, 36.6 vs. 10.2 months for patients having objective response vs. stable/progressive disease, and 38.5 vs. 14.2 months for patients having controlled vs. non-controlled disease at follow-up.

**Conclusion:**

Local control of LMBC confers a survival benefit and local interventional treatment for LMBC should be studied in a randomized trial. Patients with small metastases and limited history of systemic LMBC treatment are most likely to benefit from local approaches. Limited extrahepatic disease should not lead to exclusion from a randomized study and should not be a contraindication for local LMBC treatment as long as no randomized data are available.

**Electronic supplementary material:**

The online version of this article (doi:10.1186/s12885-015-1499-z) contains supplementary material, which is available to authorized users.

## Background

In the last two decades, the notion that formation of metastases of any malignant tumor indicates systemic spread of the disease and precludes benefit from local tumor treatment has been challenged by the observation that some patients remain disease-free after removal of their primary tumor and all visible metastatic lesions, indicating cure. As a result, surgical or locally ablative treatment of metastatic lesions is now an accepted, potentially curative modality in a variety of cancers for patients with limited metastatic burden (e.g., colorectal, renal cell, and non small-cell lung cancer) and has been integrated into treatment guidelines [1–3]. More recently, however, the distinction between “curable” and “non-curable” cancer has become less clear as some patients may continuously demonstrate controllable disease for many years and eventually die from causes unrelated to their cancer. The propensity of a cancer to develop rapid dissemination has been referred to as the disease’s biology; however, it is likely that a complex pattern of interaction between the tumor cells and the host organism rather than specific properties of the tumor alone determine the metastatic potential [4]. These observations have led to the concept of a distinct “oligometastatic” disease state which incorporates patients who may derive benefit from local treatment (even repeatedly) despite the impossibility to achieve true cure [5, 6].

It is unknown if an oligometastatic subpopulation exists among patients with metastatic breast cancer (MBC). Generally, MBC is regarded as a systemic disease and these patients, in line with current European and American treatment guidelines which essentially reserve locally ablative or surgical treatment to lesions that are symptomatic or prone to cause local complications [7, 8], are mostly offered palliative systemic treatment regardless of their metastatic burden. This is particularly true for liver metastases (LMBC) that typically occur late in the course of breast cancer and are uncommon in the absence of extrahepatic disease [9, 10]. Despite this, there are a number of retrospective, non-randomized reports demonstrating superior survival in patients undergoing liver resection for LMBC [11–15] compared to patients receiving systemic treatment alone, with surgically treated patients achieving median survival of up to 5 years and 5 year overall survival of up to 60 %, whereas median survival in MBC patients treated with systemic therapy only is approximately 24 months and only 5-10 % are alive at 5 years [16, 17]. Survival figures reported for patients treated with locally ablative modalities (radiofrequency ablation (RFA) or interstitial CT-guided brachytherapy (BT)) are generally lower, probably in part owing to a selection bias. However, they still compare favorably to systemic treatment alone, demonstrating some benefit from local control of liver metastases in a disease that is assumed to be systemic by nature [18–22]. This is further complemented by the observation that, in chemorefractory patients not amenable to surgical or locally ablative treatment, encouraging survival was observed with locoregional intrahepatic therapies (^90^Y radioembolization (RE) or intrahepatic chemotherapy) [23, 24].

As currently available data do not allow to determine if improved survival in patients undergoing locoregional therapy represents a true treatment benefit or must be regarded as an expression of favorable disease biology, with patients demonstrating relatively indolent disease preferably being selected for local treatment, it has repeatedly been suggested that these treatments be evaluated within a randomized trial comparing the combination of local and systemic therapy to systemic therapy alone. The exploratory data we present here is intended to help modelling such study concepts by identifying prognostic factors in a cohort of LMBC patients both with and without extrahepatic disease who were not amenable to radical surgery and received locally ablative (RFA or BT) or locoregional intrahepatic (RE) treatment either once or in combination as part of sequential treatment decisions.

## Methods

### Study design

This retrospective study explores prospectively collected data from patients with LMBC who were referred to our department for local interventional treatment of liver metastases between 2006 and 2010. The local ethics committee (Ethik-Kommission der Otto-von-Guericke-Universität in Magdeburg) was informed about the analyses, an approval was waived due to the retrospective nature of the study. Written informed consent for anonymized analysis of disease- and treatment-related patient data for scientific purposes was obtained from all patients.

Patients undergoing either radiofrequency ablation (RFA), interstitial catheter-based radiotherapy (BT), or ^90^Y Radioembolization (RE) for LMBC were included if: (i) imaging follow-up was available (computed tomography (CT) or magnetic resonance imaging (MRI), every 2-4 months), (ii) clinical follow-up data (including laboratory analyses) were available (every 2-4 months), (iii) a written informed consent for anonymized patient data analysis was available, (iv) no other active cancer was known.

### Patient characteristics

59 patients (58 female, 1 male, mean age 57.4 years, range 32-80) were included in this analysis. Selection of patients for local interventional treatment was based on lack of further chemotherapeutic options (progression of LMBC on all standard chemotherapeutic protocols or patient refusal of further chemotherapy) as well as surgical non-resectability of all visible lesions (either technically or due to impaired patient tolerance for major liver surgery). Patients had to have liver-predominant disease; however, limited extrahepatic disease that was stable under systemic treatment or amenable to local ablation was allowed.

The median interval from diagnosis of LMBC to presentation for interventional treatment was 22 (1-294) months.

Tumors were hormone-receptor positive in 49 of 59 patients and Her2-neu positive in 20 of 59 patients.

Differentiation of liver metastases was graded as G1 (4 patients), G2 (26 patients), or G3 (21 patients). For 8 patients no information on tumor differentiation was available.

At presentation for interventional treatment of LMBC, the mean intrahepatic tumor load (mean cumulative volume of all liver metastases relative to total liver volume) was 8.2 % (range 0.1–51.4). Mean number of liver metastases was 13 (range 1-88). The diameter of the largest liver lesion ranged from 1-14 cm (mean 4.9 cm). The mean cumulative volume of the liver metastases was 129.6 mL (range 0.5-976), and mean total liver volume was 1445 mL (range 801-2202).

54 of 59 patients had a history of 1-8 (median 2) lines of systemic chemotherapy (without hormones) for their breast cancer prior to presentation for interventional treatment of LMBC. Bisphosphonates had been applied in 24 patients. Surgery and external beam radiation for breast cancer metastases had been performed in 19 and 20 patients, respectively.

29 of 59 patients had evidence of limited extrahepatic disease at the time of interventional LMBC treatment (bone metastases only, 19/59 patients; extrahepatic disease other than bone metastases, 5/59 patients; extrahepatic disease other than bone metastases and bone metastases, 5/59 patients).

Patient and treatment characteristics are summarized in Tables 1 and 2.

**Table 1:** Baseline Characteristics (59 patients)

Sex (f/m)		58/1
Mean age (y, range)		57.4 (32 - 80)
Age ≤ 60y		63 % (*n* = 37)
Hormone receptor positive		83 % (*n* = 49)
Her2 neu positive (triple)		34 % (*n* = 20)
Grading (1/2/3/ not available)		7 % (*n* = 4)/ 44 % (*n* = 26)/36 % (*n* = 21)/13 % (*n* = 8)
Median time from first BC diagnosis to diagnosis of liver metastases (mo, range)		45 (0 – 335.4)
Median time from diagnosis of liver metastases to first interventional treatment (mo, range)		22 (1 - 294)
Mean number of liver metastases (n, range)		13 (1 - 88)
Mean maximum diameter of liver metastases (cm, range)		4.9 (1 - 14)
Mean liver volume (mL, range)		1445 (801 - 2202)
Mean volume of liver metastases (mL, range)		129.6 (0.5 - 976)
Mean tumor load (%, range)		8.2 (0.1 - 51.4)
Extrahepatic metastases		49 % (*n* = 29)
	Bone only	*n* = 19
	Bone	*n* = 24
	Lung	*n* = 6
	Lymphatic nodes (others than axillary)	*n* = 4
	Peritoneum	*n* = 1

**Table 2:** Treatment characteristics (59 patients)

Chemotherapy prior to interventional LMBC treatment (without hormones)		92 % (*n* = 54)
	median number of applied lines (range)	2 (0 - 8)
Local treatment of breast cancer metastases prior to interventional LMBC treatment, overall		68 % (*n* = 40)
	surgery (for metastasis)^a^	*n* = 19
	radiotherapy^a^	*n* = 20
Bisphophonates prior to interventional LMBC treatment		*n* = 24
Concomitant or subsequent breast cancer therapy (after initiation of interventional LMBC treatment), overall		86 % (*n* = 51)
	chemotherapy^a^	*n* = 40
	hormones^a^	*n* = 22
	surgery^a^	*n* = 7
	radiotherapy^a^	*n* = 14
Total number of interventional procedures for LMBC^a^		*n* = 97
	interstitial brachytherapy (BT)	*n* = 68
	radioembolization (RE)	*n* = 34
	radiofrequency ablation (RFA)	*n* = 5
Number of interventional procedures per patient (1/2/3/4/5/6/7) (n)		(37/13/5/0/2/1/1)
First treatment per patient (BT/RE/RFA/combination) (n)		(29/22/3/5)
Treatment modalities per patient (first and all subsequent procedures)		
	RE only	*n* = 17
	BT only	*n* = 25
	RFA only	*n* = 1
	BT and RE	*n* = 12
	BT and RFA	*n* = 3
	RE and RFA	*n* = 1

^a^more than one treatment/site per patient possible

### Locally ablative therapies

In general, BT or RFA was performed in case of no more than five hepatic metastases. RFA was preferred in patients with a maximum lesion diameter of 3 cm whereas BT was performed for lesions exceeding this limit and for lesions under 3 cm with an unfavorable location for RFA (i.e., close proximity to the liver hilum or other heat-vulnerable structures).

BT and RFA were performed under conscious sedation and analgesia using midazolam and fentanyl under continuous surveillance of vital parameters.

#### Image guided interstitial brachytherapy (BT)

The technique has been described previously [25]. Briefly, the placement of the introducer sheaths (6 F in size) with the brachytherapy applicators inside was performed under CT-fluoroscopy using Seldinger technique. For treatment planning purposes, a contrast enhanced CT of the liver was acquired. According to the defined course of the catheters, the clinical target volume and the predefined minimum dose at the tumor margin (15 Gy, delivered as a single fraction), the planning software (Oncentra, Nucletron, Veenendaal, The Netherlands) calculated a dosimetry and the dwell of the Iridium-192 source inside the brachytherapy catheters, respectively. The high-dose-rate afterloading system (Microselectron, Nucletron, Veenendaal, The Netherlands) employed an Iridium-192 source with a nominal activity of 10 Ci..

#### Radiofrequency ablation (RFA)

All RFA procedures were performed using multitined expandable electrodes (RITA Starburst; Angiodynamics, Latham, USA) that were placed under CT or MR fluoroscopy guidance. The RFA procedure was conducted according to the manufacturer’s recommendations. To control the achieved coagulation zone instantaneously after completing the procedure, a postprocedural contrast-enhanced CT scan (or a fat saturated T2-weighted spinecho sequence in case of conduction under MRI guidance) with the electrode still in place was performed.

If needed, the electrode was repositioned to achieve a volume large enough to cover the entire metastasis including a safety margin.

### Locoregional treatment

#### Radioembolization (RE)

In general, RE was performed if the number of hepatic metastases exceeded five. RE was performed employing Yttrium-90 resin microspheres (SIR-Spheres®, Sirtex Medical, Lane Cove, Australia). Treatment including pre-procedural diagnostic work-up was performed according to standard algorithm (detailed description in [26]). The activity of ^90^Y resin microspheres was calculated by the body surface area (BSA) method. ^90^Y resin microspheres were delivered selectively into the hepatic arteries (using a transfemoral approach) sequentially with an interval of 4-6 weeks between the treatments of each liver lobe (if a bilobar treatment was necessary). All patients received proton pump inhibitors (pantoprazole, 20 mg daily), low dose prednisolone (5 mg daily), and ursodeoxycholic acid (500 mg daily) for 8 weeks to ameliorate the effect of possibly migrated spheres in the gastric mucosa and the embolization effect to the liver parenchyma.

### Treatments and combinations

#### Interventional LMBC treatment

The following interventional procedures were performed: BT only (29 patients), RFA only (3 patients), RE only (22 patients). 5 Patients had localized disease in one liver lobe accompanied by multilocular metastases in the contralateral lobe and were treated with both unilateral RE and RFA or BT of the solitary contralateral lesions. 22 patients were re-treated for progressive disease following the first interventional procedure (see Table 2).

#### Further treatment

Forty patients received subsequent chemotherapy and 22 patients received hormonal therapy following interventional LMBC treatment. Surgery and external beam radiotherapy for localized extrahepatic disease were performed in 7 and 14 patients, respectively. For detailed information see Table 2.

### Imaging, volumetry and response analysis

For all patients a baseline Gd-EOB-DTPA (Primovist, Bayer Healthcare, Leverkusen, Germany, 0.025 mmol/kg/bodyweight) enhanced MRI of the liver was available. Baseline MRI (hepatobiliary phase T1- weighted imaging, 5 mm slice thickness) was used for volumetry of the liver and tumor volume as well as for measurement of the tumor diameters using the image processing software Osirix (Antoine Rosset, 2003-2011).

Follow-up imaging consisted of either MRI of the liver or CT of the abdomen (with or without thorax) every 2-4 months. MRI (1.5 Tesla system, Achieva 1.5 T, Philips, Best, The Netherlands) of the liver was conducted using Gd-EOB-DTPA as i.v. contrast agent. For response analysis hepatobiliary phase imaging (T1-weighted imaging, 5 mm slice thickness) was used. In case of inconclusiveness, other sequences (contrast dynamic, T2-weighted imaging) were taken into account. CT (multislice CT, either 16 (Toshiba Aquilion, Toshiba Medical, Tokyo, Japan) or 64 (Siemens Definition AS, Erlangen, Germany) row detector system) was conducted using 90 mL iodinated contrast media (Imeron 300, Iomeprol, Bracco, Princeton, USA) with a reconstructed slice thickness of 5 mm. For response analysis, venous phase imaging was used.

Response analysis according to RECIST 1.0 was performed separately for the liver only and overall. Analyses of response to treatment are based on best response recorded during follow-up.

As an additional efficacy descriptor beyond RECIST, control of LMBC by interventional treatment during follow-up was used, with patients demonstrating either overall objective response, stable disease, or limited disease progression amenable to repeat local ablation being regarded as having controlled disease. Disease progression not amenable to local intervention was defined as non-controlled.

### Toxicity analysis

All patients underwent standard clinical and laboratory examination including liver-related parameters at first presentation and during follow up after interventional treatment. The Common Terminology Criteria for Adverse Events (CTCAE) version 4.02 (National Cancer Institute, USA) were used for toxicity assessments of laboratory values and clinical findings.

### Statistics

Statistical analysis was performed using SPSS (SPSS 21, IBM, Chicago, Il, USA). Descriptive analysis of patient characteristics and findings was performed with continuous variables displayed as mean or median with standard deviation or range and frequency data displayed as counts.

Survival (from first diagnosis, first diagnosis of liver metastases, first interventional treatment of LMBC) was estimated according to the Kaplan-Meier method.

Possible factors influencing survival were included in a univariate Cox model. Continuous variables were dichotomized according to a ROC analysis using survival (longer vs. shorter than median overall survival) as the target variable. Optimal cut-off was determined according to the Youden index (see Additional file 1: Table S1).

Interactions of variables found to have significant influence on survival in the univariate analysis were evaluated by the Chi-square test/Fisher’s exact test (see Additional file 2: Table S2). In case of interactions, either the variable with the lower p-value in the univariate Cox model or the more practicable variable was chosen. To test the independent impact of each confounder identified at univariate analysis on survival, multivariate Cox models were created including all variables that were unevenly distributed between the groups of patients with shorter vs. longer than median survival and did not show significant interaction. This analysis was first performed excluding parameters that were not available prior to treatment and then repeated using all parameters (including those available at follow-up only). Additionally, due to interaction of these variables, separate models were created to analyze the survival impact of either overall extrahepatic disease or bone metastases as the sole extrahepatic tumor manifestation.

Factors found to have an independent impact on survival in the multivariate model were used as stratifying variables in a Kaplan-Meier analysis of survival after first interventional LMBC treatment. The log rank test was used for survival comparison.

A *p* < 0.05 was considered statistically significant.

## Results

Median overall survival from first interventional treatment of LMBC was 21.9 months (95 % CI: 11.1-32.6), from first diagnosis of liver metastases, 56.3 months (44.5-67.9), and from first diagnosis of breast cancer, 127.9 months (87.1-168.7) (Table 3).

**Table 3:** Survival and Progression^a^

		months (median)		95 % CI		*p*-value
Follow-up		16.14				
Overall survival						
	from first diagnosis	127.9		87.1- 168.7		
	from first diagnosis liver metastases	56.3		44.5 - 67.9		
	from first interventional treatment	21.9		11.1 - 32.6		
Maximum diameter of liver metastases (≥ vs. < 3.9 cm)		16.1	38.7	9.8 - 22.5	19.6 - 57.8	0.001
Best response overall (OR, RECIST)						
	OR (CR + PR) (*n* = 37) vs. PD + SD (*n* = 22)	36.6	10.2	26.4 - 46.7	6.1 - 14.3	< 0.001
Disease controlled during follow-up (yes/no)^b^		38.5	14.2	27.1 - 39.7	9.4 - 18.9	0.002

Survival (from first diagnosis, first diagnosis of liver metastases, first interventional treatment of LMBC) estimated according to the Kaplan-Meier method. Factors found to have an independent impact on survival in the multivariate model (Table 5) were used as stratifying variables in a Kaplan-Meier analysis of survival after first interventional LMBC treatment. The log rank test was used for survival comparison

^a^Addressing overall survival data and significant influencing factors from multivariate cox regression

^b^Patients demonstrating either overall objective response, stable disease, or limited disease progression amenable to repeat local ablation were regarded as having controlled disease. Disease progression not amenable to local intervention was defined as non-controlled

The ROC analysis used to dichotomize continuous variables with respect to median overall survival yielded the following significant results: number of liver metastases (optimal cut-off: </≥ 6 lesions), maximum diameter of liver metastases (</≥ 3.9 cm), volume of liver metastases (</≥ 27.9 mL), intrahepatic tumor load (</≥ 2 %), liver volume (</≥ 1376 mL), number of previous lines of chemotherapy (</≥ 3), CA 15-3 (</≥ 62.6U/mL), and CEA (</≥ 6.2U/mL) (see Additional file 1: Table S1). Dichotomized variables were used for further analyses. Although the ROC analysis was unable to dichotomize time from first breast cancer diagnosis to diagnosis of liver metastases, this variable was still considered a possible influencing factor for further analyses with a cut-off based on data reported in the literature (<2 years vs. ≥ 2 years; [27]).

Univariate Cox regression regarding overall survival from first interventional LMBC treatment of the LMBC yielded type of initial local treatment (BT/RFA vs. RE), presence of extrahepatic metastases, presence of bone metastases only, number of liver metastases, maximum diameter of liver metastases, volume of liver metastases, tumor load, liver volume, number of lines of chemotherapy applied prior to interventional LMBC treatment, CA 15-3, CEA, best response during follow-up (overall), best response during follow-up (liver only), and control of LMBC by interventional treatment during follow-up (according to the definition given in the methods section) as factors with a significant survival impact (Table 4). These variables were then tested for inter-variable interaction (see Additional file 2: Table S2) and the following non-interacting variables were extracted for inclusion into the multivariate Cox models: presence of extrahepatic metastases (and presence of bone metastases only, separate model), liver volume, maximum diameter of liver metastases, number of liver metastases, number of lines of chemotherapy applied prior to interventional LMBC treatment, control of LMBC by interventional treatment during follow-up, and best overall response to interventional treatment during follow-up. When only factors available at treatment initiation were included in the analysis, only maximum diameter of liver metastases (≥ 3.9 cm; HR: 3.1), liver volume (≥ 1376 mL; HR: 2.3), and history of prior chemotherapy (≥ 3 lines of treatment; HR: 2.5-2.6) showed a significant impact on survival (Table 5a). Analyzing all potential factors including those available at follow-up only, only maximum diameter of liver metastases (≥ 3.9 cm; HR: 3.1), control of LMBC by interventional treatment during follow-up (HR: 0.29), and objective response as best overall response during follow-up (HR: 0.21) demonstrated a significant influence on survival (Table 5b). Neither the presence of any extrahepatic metastases nor presence of bone metastases only had a significant impact on survival in any of the models (Table 5a and b).

**Table 4:** Univariate Cox Regression for Survival

Variable		Hazard ratio	95 % CI	*p*-value
Age		0.99	0.97 - 1.03	0.836
Age (>60y)		0.51	0.24 - 1.11	0.089
Hepatic progression in FU (yes)		0.8	0.37 - 1.73	0.566
Systemic progression in FU (yes)		1.57	0.78 - 3.17	0.209
Under local control in FU (yes)		0.31	0.14 - 0.68	0.004
Her2 neu (pos)		1.22	0.59 - 2.53	0.588
Hormone receptor (pos)		0.96	0.41 - 2.24	0.926
Grading (1-3)		0.93	0.5 - 1.73	0.826
Other prior therapies for metastastes (yes)		1.99	0.89 - 4.43	0.094
Extrahepatic metastases (yes)		2.86	1.39 - 5.87	0.004
	bones only	3.01	1.49 - 6.08	0.002
First local treatment				
	Brachytherapy (BT) or RFA	0.28	0.13 - 0.59	0.001
	Radioembolization (RE)	3.31	1.57 - 6.98	0.002
	Combination RE/BT or RE/RFA	1.14	0.40 - 3.26	0.813
Best response hepatic (OR, RECIST)		0.37	0.17 - 0.79	0.01
Best response overall (OR, RECIST)		0.15	0.07 - 0.34	< 0.001
Clinical and laboratory grade 3/4 toxicities (yes)		1.83	0.78 - 4.31	0.168
Number of liver metastases (≥ 6)		2.08	1.01 - 4.31	0.048
Maximum diameter of liver metastases (≥ 3.9 cm)		3.43	1.57 - 7.52	0.002
Liver volume (≥ 1376 mL)		2.27	1.11 - 4.64	0.024
Volume of liver metastases (≥ 27.9 mL)		4.33	1.91 - 9.79	< 0.001
Tumor load (≥ 2 %)		5.6	2.37 - 13.23	< 0.001
Lines of chemotherapy (≥ 3)		3.17	1.55 - 6.49	0.002
CA 15-3 (≥ 62.6U/mL)		2.42	1.10 - 5.36	0.029
CEA (≥ 6.2U/mL)		3.36	1.58 - 7.15	0.002
Time from first diagnosis to liver metastases (≥ 2y)		0.79	0.40 - 1.56	0.492
Concomitant or subsequent therapies for breast cancer metastases (yes)		0.43	0.16 - 1.16	0.094

Possible factors influencing survival were included in a univariate Cox model. Continuous variables were dichotomized according to a ROC analysis (see Additional file 1: Table S1)

**Table 5:** Multivariate Cox Regression for Survival

a. Multivariate Cox Regression for Survival, Factors available at treatment initiation only			
Variable set	Hazard ratio	95 % CI	p-value
Extrahepatic metastases (yes)	2.13	0.97 - 4.64	0.058
Liver volume (≥ 1376 mL)	2.25	1.01 - 5.02	0.047
Maximum diameter of liver metastases (≥ 3.9 cm)	3.12	1.39 - 7.02	0.006
Number of liver metastases (≥ 6)	1.48	0.69 - 3.14	0.312
Lines of chemotherapy (≥ 3)	2.48	1.15 - 5.36	0.021
Bone metastases only (yes)	1.56	0.70 - 3.47	0.279
Liver volume (≥ 1376 mL)	2.01	0.93 - 4.36	0.076
Maximum diameter of liver metastases (≥ 3.9 cm)	3.1	1.37 - 7.02	0.007
Number of liver metastases (≥ 6)	1.51	0.72 - 3.20	0.278
Lines of chemotherapy (≥ 3)	2.6	1.14 - 5.92	0.023
b. Multivariate Cox Regression for Survival, All Factors (Pre- and Posttherapeutic)			
Variable set	Hazard ratio	95 % CI	p-value
Extrahepatic metastases (yes)	1.05	0.43 - 2.58	0.92
Liver volume (≥ 1376 mL)	2.17	0.90 - 5.22	0.084
Maximum diameter of liver metastases (≥ 3.9 cm)	3.1	1.31 - 7.36	0.01
Number of liver metastases (≥ 6)	1.13	0.49 - 2.59	0.782
Lines of chemotherapy (≥ 3)	1.81	0.83 - 3.97	0.138
Under local control in FU (yes)	0.29	0.11 - 0.73	0.009
Best response overall (OR, RECIST)	0.21	0.08 - 0.57	0.002
Bone metastases only (yes)	1.06	0.46 - 2.44	0.89
Liver volume (≥ 1376 mL)	2.16	0.92 - 5.03	0.075
Maximum diameter of liver metastases (≥ 3.9 cm)	3.05	1.22 - 7.61	0.017
Number of liver metastases (≥ 6)	1.12	0.48 - 2.59	0.796
Lines of chemotherapy (≥ 3)	1.8	0.80 - 4.01	0.154
Under local control in FU (yes)	0.29	0.12 - 0.70	0.006
Best response overall (OR, RECIST)	0.21	0.08 - 0.52	0.001

Multivariate cox models were created on the basis of significant and non-interacting factors from univariate analysis with one model exclusively analyzing factors available prior to treatment (5 a.) and a separate model including all factors including those available at follow-up only (5 b.). Additionally, due to interaction of these variables, separate models were created to analyze the survival impact of either overall extrahepatic disease or bone metastases as the sole extrahepatic tumor manifestation

Overall survival estimates stratified for the maximum diameter of liver metastases (</≥ 3.9 cm) were 38.7 vs. 16.1 months (*p* = 0.001), for best overall response during follow-up (objective response vs. SD and PD), 36.6 vs. 10.2 months (*p* < 0.001), and for control of LMBC by interventional treatment during follow-up (yes vs. no), 38.5 vs. 14.2 months (*p* = 0.002) (Table 3).

After interventional treatment of LMBC, 9 grade 3 and no grade 4 toxicities were recorded. This included elevation of alanine aminotransferase (1 patient), elevation of alkaline phosphatase and reduction of albumin (1 patient), liver decompensation (defined as ascites unrelated to disease progression; 5 patients), and i.v. port infection (1 patient). All reported toxicities occurred after radioembolization. Patients developing any grade 3 toxicity following treatment had a tendency towards decreased survival (median survival, 8.2 vs. 14.2 months with vs. without toxicity in patients receiving RE at any time during their treatment sequence); however, this was not significant (*p* = 0.234).

## Discussion

In the past, scientific workup of surgical treatment for patients with metastatic cancers has been hampered by the fact that surgery used to carry substantial risks which were considered to be non-justified in a disease that is unlikely to be cured. Nonetheless, with the continuing improvement of perioperative outcomes in liver surgery, there is now an increasing role for liver resection as part of a multidisciplinary treatment concept in many cancers, even outside of a curative approach [28, 29]. Minimally invasive local treatment modalities have been developed to further reduce periprocedural risks. Recent data referring to a variety of techniques demonstrates encouraging results regarding local tumor clearance, making them particularly attractive in patients in whom a more radical approach associated with higher morbidity may not be acceptable because a survival benefit cannot be reliably predicted. Among these treatments, RFA is the most widely accepted modality and is regularly used to ablate liver tumors of limited number and size (usually <3-5 cm) in a suitable location (i.e., outside the immediate vicinity of large vessels to minimize the heat-sink effect, or at some distance from the hepatic bifurcation) [30]. Image-guided, catheter-based, interstitial brachytherapy is employed by an increasing number of centers since it avoids the tumor size limitation applicable for RFA [20, 25, 31]. Finally, RE using ^90^Y-labelled glass or resin microspheres is able to target multifocal lesions not amenable to RFA or interstitial BT due to its inherent locoregional effects. RE has demonstrated impressive response rates in both primary and metastatic liver tumors [32].

No data is currently available on the outcome of single, combined or sequential use of such locally and locoregionally active devices. For the cohort described herein, treatment decisions were generally based on 2 dominant terms:

- a therapeutic algorithm mirroring the individual patient situation, i.e., all disease except for (controlled) bone metastases was amenable to treatment using a minimally invasive technique; systemic chemotherapy was either not effective, not further applicable due to toxicity or refused by the patient.
- the selection of the appropriate device followed the technical considerations described above; i.e., RFA for patients with up to 3 tumors up to 3 cm in diameter (if not adjacent to main portal vein or hepatic bifurcation); BT for patients with up to 5 tumors any size in any location; RE for diffuse disease with up to 50 % tumor load.

Due to its liberal inclusion criteria with no upper limit to the number and size of liver metastases, extensive prior chemotherapy in almost all patients, and inclusion of patients with controlled extrahepatic disease (both sceletal and extrasceletal), the present study represents a negatively selected cohort of patients. In this dismal patient selection with LMBC diagnosed almost 2 years (median 22 months) prior to presentation for local treatment, the observed median overall survival of 21.9 months compares favorably with literature results on patients with LMBC undergoing systemic treatments only [33]. Our patient cohort recruited between 2006 and 2010 did not benefit of advances in systemic treatments that were recently reported for the use of nab-Paclitaxel in patients with metastatic breast cancer [34], with approx. 80 % of the patients in that study having visceral metastases whereas the proportion of hepatic metastases (that are considered to confer a particularly poor prognosis [35]) was not specified. Hence, our results must be compared to studies employing anthracycline, taxanes or cyclophosphamide. Data available refers to first line patients (in contrast to our cohort in a salvage situation), and median overall survival ranged from 22.7 to 27.1 months in patients with liver metastases only and from 14-16.8 months in patients with liver-dominant and limited extrahepatic disease [10, 9, 36]. The beneficial effect of a local treatment approach is further complemented by the observation that local control of the liver metastases and objective response to locoregional treatment at follow-up were found to be the strongest factors indicating prolonged survival at multivariable analysis in our study (HR of 0.29 and 0.21, respectively). Thus, in contrast to the view held by some investigators that local control of liver metastases in breast cancer has no survival impact due to the generalized nature of the disease [37], our results (in line with other published research [38]) are rather suggestive of the existence of an oligometastatic subpopulation in LMBC patients whose prognosis is determined by the visible lesions rather than subclinical tumor dissemination which will cause rapid systemic progression regardless of local disease control. The observation that most patients developing recurrence following resection of LMBC have the liver as their first site of recurrence further underscores this view [39, 40].

There are currently no methods to reliably identify the oligometastatic subpopulation which may benefit from local surgical or image-guided treatment approaches. In the absence of established molecular biomarkers, the selection of metastatic breast cancer patients who are most likely to derive benefit relies on retrospective identification of prognostic factors in patients receiving these therapies. In published studies, patient characteristics most often reported to be associated with prolonged survival include long disease-free interval between the treatment of the primary cancer and the diagnosis of liver metastases [40, 41], small size and/or low number of liver metastases [14, 15, 21, 42], well-differentiated histopathology [14], and response to pre-interventional or preoperative chemotherapy [43]. In some studies, patients demonstrating extrahepatic disease were generally excluded from local or locoregional treatment [14, 42, 44]. In the studies that did include patients with tumor spread beyond the liver, presence of extrahepatic disease was identified as a poor prognostic factor by some [12, 13, 45] but not all investigators [21, 43]. In the study by Jakobs et al. [22], presence of extrahepatic disease in patients undergoing RFA of liver metastases was associated with poor prognosis; however, this did not apply to patients who had bone metastases as their only extrahepatic tumor site.

Since factors predictive for treatment benefit should be available before treatment is started, we first analyzed the survival impact of parameters prior to treatment only. Of these parameters, only large liver volume, tumor diameter of ≥ 3.9 cm, and at least 3 lines of chemotherapy prior to local treatment were associated with poor prognosis. Presence of extrahepatic disease showed a tendency towards a negative prognostic impact (*p* = 0.058) when only factors known prior to the therapeutic decision were included. The negative effect almost vanished if extrahepatic disease was limited to bone metastases only. To evaluate the survival benefit conferred by locally ablative treatment, parameters related to treatment outcome that were available at follow-up only were entered into a subsequent analysis. Sustained local control of the liver lesions and objective treatment response were the strongest predictors of good outcome in this setting, whereas neither the presence of any extrahepatic disease, nor evidence of bone metastases as the only extrahepatic tumor site preserved their tendency towards a negative prognostic impact.. Interestingly, the impact of prior chemotherapy was also no longer significant, indicating that even heavily pre-treated patients may derive benefit from locoregional treatment modalities if local control of the liver lesions is achieved.

The treatments used in our study appeared to be generally safe, with no grade 4 toxicities observed and only few grade 3 toxicities occurring exclusively among patients treated with RE. Although a tendency towards decreased survival in patients developing grade 3 toxicity was noted, this was not significant. A larger analysis is required to reliably establish the relationship between treatment toxicity and outcome, and to determine how toxicities occurring after locally ablative treatment impact patient’s tolerance for further antineoplastic therapy.

In summary, our results indicate that patients most likely to derive benefit from local or locoregional LMBC treatment are those with a largest tumor diameter of < 4 cm and only limited history of systemic chemotherapy prior to treatment. According to our data, patients with controlled extrahepatic disease, and specifically patients with bone metastases as the only extrahepatic site, may be considered for local ablation. We postulate that this is the patient population that should be selected for future trial concepts designed to compare local tumor treatment plus systemic chemotherapy with standard chemotherapy alone.

Our study has several limitations. First, we have no sound explanation for the finding that liver volume ≥1376 ml was identified as an independent poor prognostic factor. Because liver volume included the volume of any intrahepatic tumor lesions, one might suspect that this was simply an expression of high tumor load being responsible for increased liver volume, thereby impairing survival. However, there was no correlation between liver volume and the volume of liver metastases (see Additional file 2: Table S2). For patients undergoing RE, a possible explanation could be that dose calculation according to the BSA method as in our study may lead to relative underdosing of ^90^Y in patients whose liver volume to BSA ratio is elevated compared to the average population. Using our data, we were unable to test this hypothesis and any attempt at providing an explanation for this finding must remain speculative. Second, our selection of patients based on the criterion “no uncontrolled extrahepatic disease” was somewhat subjective. This represents clinical practice at our institution as well as in many other centers. It may be appropriate as long as neither a broadly accepted definition of “controllable disease” nor treatment guidelines exist. However, for a future prospective trial a clear definition of what extent of extrahepatic disease is acceptable must be adopted. According to our data, this should clearly include patients with bone metastases only. For extrasceletal metastases, a possible threshold could be sustained disease stabilization for at least 12 months prior to local treatment as in the study by Hoffmann et al. [40]. Third, with patients included in our study who underwent RFA, interstitial brachytherapy, and RE, a very heterogeneous spectrum of treatment modalities and tumor distribution within the liver was analyzed. Thermal (RFA) vs. non-thermal (BT and RE) treatments are based on different working principles, as are high-dose (BT) vs. low-dose (RE) radiation therapy. If high-dose, catheter-based brachytherapy or low-dose, microsphere-based radioembolization is more effective in terms of response as well as recurrence rate and survival will be an area of high interest for larger, future analyses. The aim of our study, however, was to obtain information on the general benefit of liver-directed local treatment in metastatic breast cancer and to identify predictive and prognostic factors to help modelling future research strategies. All of the treatments studied exclusively target intrahepatic disease; hence, we considered it appropriate for our exploratory purposes to perform a joint analysis of the results obtained with all of these treatments.

## Conclusions

In conclusion, our results confirm that patients with hepatic metastases from breast cancer, despite being incurable in most cases, may have favorable survival outcomes from locoregional treatment of their liver disease. The exact role of such therapies must be established in a randomized trial. Our study supports the assumption that an “oligometastatic” subgroup exists among patients with breast cancer metastases and that, as long as no reliable biomarkers exist to predict disease behavior, even limited extrahepatic disease should not automatically exclude patients from being treated locally.

## Additional files

Additional file 1: Table S1. ROC Analysis and Cut-off Values. In order to prepare continuous variables for the Cox model, the according variables were dichotomized by a ROC analysis using survival (longer vs. shorter than median overall survival) as the target variable. Optimal cut-off was determined according to the Youden index. Variables with no significant cut-off were not used for the cox model, except the variable time from first breast cancer diagnosis to diagnosis of liver metastases since this variable was still considered as a possible influencing factor according to literature (with a cut off <2 years vs. ≥ 2 years).
Additional file 2: Table S2. Chi-Square Test for Interactions. The Chi-Square test was used to identify interactions between variables with influence on survival according to the univariate cox model in order to build up a robust multivariate cox model without interacting variables.
