# Supplementary material for: Locally ablative treatment of breast cancer liver metastases: identification of factors influencing survival (the Mammary Cancer Microtherapy and Interventional Approaches (MAMMA MIA) study)
Source: BMC Cancer. 2015 Jul 14;15:517. doi: 10.1186/s12885-015-1499-z (PMC4501116; doi:10.1186/s12885-015-1499-z)
Supplement: Additional file 1: Table S1. — ROC Analysis and Cut-off Values. In order to prepare continuous variables for the Cox model, the according variables were dichotomized by a ROC analysis using survival (longer vs. shorter than median overall survival) as the target variable. Optimal cut-off was determined according to the Youden index. Variables with no significant cut-off were not used for the cox model, except the variable time from first breast cancer diagnosis to diagnosis of liver metastases since this variable was still considered as a possible influencing factor according to literature (with a cut off <2 years vs. ≥ 2 years). [file 12885_2015_1499_MOESM1_ESM.docx]

Additional file, Table S1

|  |  |  |  |  |  |  |  |  |
| --- | --- | --- | --- | --- | --- | --- | --- | --- |
| ROC Analysis and Cut-off Values | | | | | | | | |
|  |  |  |  |  |  |  |  |  |
|  |  | AUC | p-value | optimal cut-off | cases (n) < ≥ of cut-off | Sensitivity | Specificity | Youden-Index |
|  |  |  |  |  |  |  |  |  |
| Age (y) |  | 0.484 | 0.839 | n.a. | n.a. | n.a. | n.a. | n.a. |
| Pre-therapeutic bilirubin (μmol/l) | | 0.445 | 0.485 | n.a | n.a. | n.a. | n.a. | n.a. |
| Number of liver metastases (n) | | 0.724 | **0.004** | < ≥ 6 | 27/32 | 0.676 | 0.682 | 0.994 |
| Maximum diameter of liver metastases (cm) | | 0.675 | **0.026** | < ≥ 3.9 | 28/31 | 0.622 | 0.636 | 0.985 |
|  |  |  |  |  |  |  |  |  |
| Liver volume (mL) | | 0.721 | **0.005** | < ≥ 1376 | 27/32 | 0.73 | 0.763 | 0.957 |
| Volume of liver metastases (mL) | | 0.742 | **0.002** | < ≥ 27.9 | 27/32 | 0.676 | 0.682 | 0.994 |
| Tumor load (%) | | 0.715 | **0.006** | < ≥ 2 | 27/32 | 0.703 | 0.727 | 0.975 |
| Lines of chemotherapy (n) | | 0.714 | **0.006** | < ≥ 3 | 33/26 | 0.541 | 0.727 | 0.813 |
| CA 15_3 (U/mL) | | 0.714 | **0.011** | < ≥ 62.6 | 24/27 | 0.594 | 0.632 | 0.962 |
| CEA (U/mL) | | 0.837 | **< 0.001** | < ≥ 6.2 | 26/33 | 0.735 | 0.762 | 0.973 |
| Time from first diagnosis to liver metastases (months) | | 0.467 | 0.672 | n.a. | n.a. | n.a. | n.a. | n.a. |
|  |  |  |  |  |  |  |  |  |
|  |  |  |  |  |  |  |  |  |

In order to prepare continuous variables for the cox model, the according variables were dichotomized by a ROC analysis using survival (longer vs. shorter than median overall survival) as the target variable. Optimal cut-off was determined according to the Youden index. Variables with no significant cut-off were not used for the cox model, except the variable time from first breast cancer diagnosis to diagnosis of liver metastases since this variable was still considered as a possible influencing factor according to literature (with a cut off <2 years vs. ≥ 2 years).
