# Supplementary material for: Locally ablative treatment of breast cancer liver metastases: identification of factors influencing survival (the Mammary Cancer Microtherapy and Interventional Approaches (MAMMA MIA) study)
Source: BMC Cancer. 2015 Jul 14;15:517. doi: 10.1186/s12885-015-1499-z (PMC4501116; doi:10.1186/s12885-015-1499-z)
Supplement: Additional file 2: Table S2. — Chi-Square Test for Interactions. The Chi-Square test was used to identify interactions between variables with influence on survival according to the univariate cox model in order to build up a robust multivariate cox model without interacting variables. [file 12885_2015_1499_MOESM2_ESM.docx]

Additional file, Table S2

|  |  |  |  |
| --- | --- | --- | --- |
| Chi-Square Test for Interactions | | | |
|  |  |  |  |
| Pairs of variables | | | p-value |
| Tumor load to maximum size of liver metastases | | | **< 0.001** |
| Tumor load to number of liver metastases | | | **0.015** |
| Tumor load to liver metastases volume | | | **< 0.001** |
| Tumor load size, number and volume of metastases to liver volume | | | >0.05 each |
| Number of liver metastases to liver metastases volume | | | **0.015** |
| Number of liver metastases to maximum size of liver metastases | | | 0.095 |
| Liver metastases volume to maximum size of liver metastases | | | **< 0.001** |
| Extrahepatic metastases to bone metastases | | | **< 0.001** |
| Bone metastases to bone metastases only | | | **< 0.001** |
| Bone metastases only to extrahepatic metastases | | | **< 0.001** |
| CEA to CA 15-3 |  |  | **0.004** |
| CEA to tumor load, size, number and volume of metastases | | | **< 0.05 each** |
| BT or RFA to tumor load, size, number and volume of metastases* | | | **< 0.05 each** |
| RE to tumor load, size, number and volume of metastases | | | **< 0.05 each** |
| Best response liver to best response overall | | | **< 0.001** |
| Best response overall to under local control in FU | | | 0.13 |
| Best response liver to under local control in FU | | | 0.613 |
| * inverse correlation |  |  |  |

The Chi-Square test was used to identify interactions between variables with influence on survival according to the univariate cox model in order to build up a robust multivariate cox model without interacting variables.
